# Supplementary material for: The relationship between spatial configuration and functional connectivity of brain regions
Source: eLife. 2018 Feb 16;7:e32992. doi: 10.7554/eLife.32992 (PMC5860869; doi:10.7554/eLife.32992)
Supplement: Supplementary file 1. — (a) Highly similar associations between behaviour and the brain can be found across a wide range of different measures derived from fMRI. We included a set of network matrices, spatial maps and amplitudes (node timeseries standard deviation) derived from several distinct group-average spatial parcellations/decompositions: from ICA decompositions at two scales of detail (dimensionalities of 25 and 200); a PROFUMO decomposition (PFM; dimensionality 50); an atlas-based hard parcellation (108 parcels [Yeo et al., 2011]); task contrast spatial maps (86 contrasts); and MSM warp fields from native space to MSMAll aligned data (from estimate_metric_distortion; https://github.com/ecr05/MSM_HOCR_macOSX/blob/master/src/MSM/estimate_metric_distortion.cc). Each row reports a separate CCA analysis, performed against behaviour/life-factors. A very similar mode of variation is found across most of the parcellation methods and different fMRI measures. rU-V is the strength of the canonical correlation between imaging and non-imaging measures (confidence intervals estimated using surrogate data), PU-V is the associated (family-wise error corrected) p-value estimated using permutation testing, taking into account family structure, and rU-V CI is the 2.5–97.5% confidence interval estimated using surrogate data. rU-Uica is the correlation of a CCA mode (subject weights) with the positive-negative mode of population covariation obtained from ICA200 partial network matrices as used in Smith et al. (2015), and is therefore defined to be one in the row containing the results from that CCA. The rU-Uica result was included because it shows whether different metrics are associated with similar or distinct behavioural modes of population covariation (one may expect different rfMRI measures to be associated with distinct aspects of behaviour). The final column contains the total number of CCA modes with PU-V <0.05 (results in other columns correspond to the most significant CCA mode, except for r [file elife-32992-supp1.docx]

## Supplementary File 1

**Supplementary file 1a**

|  |  | **r_U-V_** | **r_U-V_ CI**  **2.5-97.5%** | **P_U-V_** | **r_U-Uica_** | **# CCAs p<0.05** |
| --- | --- | --- | --- | --- | --- | --- |
| **ICA**  **d=25**  **N=819** | Full correlation network matrix  Partial correlation network matrix  Amplitudes  Spatial maps | 0.73  0.72  0.56  0.77 | 0.71-0.75  0.71-0.74  0.54-0.59  0.76-0.80 | 0.00001  0.00001  0.00002  0.00001 | 0.43  0.42  0.37  0.78 | 3  2  2  3 |
| **ICA**  **d=200**  **N=819** | Full correlation network matrix  Partial correlation network matrix  Amplitudes  Spatial maps | 0.73  0.79  0.72  0.77 | 0.72-0.75  0.78-0.82  0.70-0.74  0.75-0.79 | 0.00001  0.00001  0.00001  0.00001 | 0.56  ≜1  0.64  0.78 | 3  3  1  2 |
| **PFM**  **d=50**  **N=819** | Full correlation network matrix  Partial correlation network matrix  Amplitudes  Spatial maps | 0.67  0.67  0.69  0.80 | 0.66-0.69  0.65-0.69  0.67-0.71  0.78-0.82 | 0.00482  0.01774  0.00006  0.00001 | 0.31  0.34  0.29  0.81 | 1  1  1  3 |
| **Yeo**  **d=108**  **N=819** | Full correlation network matrix  Partial correlation network matrix  Amplitudes | 0.73  0.77  0.67 | 0.71-0.75  0.76-0.79  0.65-0.68 | 0.00001  0.00001  0.05546 | 0.60  0.69  0.37 | 2  3  0 |
| **Task**  **N=790** | Contrast spatial maps (d=86) | 0.81 | 0.79-0.83 | 0.00001 | 0.57 | 1 |
| **Warp N=819** | Warp field from native space to MSMAll alignment | 0.72 | 0.70-0.74 | 0.00001 | 0.53 | 1 |

**Supplementary file 1b**

|  |  | **r_U-V_** | **r_U-V_ 5% CI** | **P_U-V_** | **r_U-Uica_** |
| --- | --- | --- | --- | --- | --- |
| **PFM**  **d=50**  **N=441** | Full correlation network matrix  Partial correlation network matrix  Amplitudes  Spatial maps | 0.84  0.86  0.85  0.88 | 0.83-0.86  0.85-0.87  0.84-0.86  0.86-0.89 | 0.06733  0.00096  0.03058  0.00002 | 0.26  0.20  0.25  0.79 |
| **HCP_MMP1.0**  **d=360**  **N=441** | Full correlation network matrix  Partial correlation network matrix  Amplitudes  Spatial maps | 0.88  0.88  0.86  0.87 | 0.87-0.89  0.87-0.90  0.85-0.88  0.86-0.89 | 0.00001  0.00001  0.00066  0.00022 | 0.41  0.59  0.33  0.51 |
| **Area N=441** | Fractional surface area for all parcels in HCP_MMP1.0 parcellation | 0.87 | 0.86-0.89 | 0.00003 | 0.40 |

###

###

### Supplementary file 1c

| **Full network matrices** | **Results driven by subject variability in:** | **Network matrix** | **Amplitude** | **Spatial map** | **Z _network matrix_** | **R _correlation_** | **CCA r _U-V_** | **CCA P _U-V_** | **CCA r _U-Uica_** |
| --- | --- | --- | --- | --- | --- | --- | --- | --- | --- |
| ICA  D = 200  N=819 | Nothing  Amps & spatial  Connectivity only  Amplitudes only  Spatial maps only | -  -  **✓**  -  - | -  **✓**  -  **✓**  - | -  **✓**  -  -  **✓** | -0.0003  1.14  0.47  0.22  0.78 | 0.03  0.60  0.65  0.15  0.54 | 0.65  0.71  0.69  0.69  0.72 | 0.32017  0.00001  0.00028  0.00052  0.00001 | 0.11  0.52  0.40  0.45  0.62 |
| ICA  D = 25  N=819 | Nothing  Amps & spatial  Connectivity only  Amplitudes only  Spatial maps only | -  -  **✓**  -  - | -  **✓**  -  **✓**  - | -  **✓**  -  -  **✓** | -0.0004  1.19  0.88  0.26  0.78 | -0.003  0.52  0.75  0.08  0.61 | 0.65  0.71  0.69  0.67  0.73 | 0.26790  0.00001  0.00005  0.03876  0.00001 | 0.12  0.47  0.44  0.47  0.47 |
| Yeo parcellation  D = 109  N=819 | Nothing  Amps & spatial  Connectivity only  Amplitudes only  Spatial maps only | -  -  **✓**  -  - | -  **✓**  -  **✓**  - | -  **✓**  -  -  **✓** | -0.002  1.09  0.50  0.25  0.69 | -0.01  0.40  0.55  0.10  0.40 | 0.65  0.72  0.69  0.68  0.74 | 0.14899  0.00001  0.00003  0.00258  0.00001 | 0.14  0.59  0.47  0.27  0.61 |
| HCP_MMP1.0 subject parcellation  D = 360  N=441 | Nothing  Amps & spatial  Connectivity only  Amplitudes only  Spatial maps only | -  -  **✓**  -  - | -  **✓**  -  **✓**  - | -  **✓**  -  -  **✓** | 0.19  1.04  0.46  0.30  0.65 | 0.19  0.38  0.62  0.14  0.45 | 0.85  0.86  0.85  0.84  0.85 | 0.02565  0.00055  0.01399  0.17321  0.01161 | 0.34  0.51  0.27  0.34  0.70 |
| HCP_MMP1.0 group parcellation  D = 360  N = 441 | Nothing  Amps & spatial  Connectivity only  Amplitudes only  Spatial maps only | -  -  **✓**  -  - | -  **✓**  -  **✓**  - | -  **✓**  -  -  **✓** | -0.0005  1.03  0.43  0.21  0.65 | -0.01  0.41  0.62  0.11  0.43 | 0.85  0.86  0.86  0.84  0.86 | 0.01726  0.00153  0.00034  0.18253  0.00850 | 0.18  0.48  0.37  0.29  0.61 |

**Supplementary file 1d**

| **Partial network matrices** | **Results driven by subject variability in:** | **Network matrix** | **Amplitude** | **Spatial map** | **Z _network matrix_** | **R _correlation_** | **CCA r _U-V_** | **CCA P _U-V_** | **CCA r _U-Uica_** |
| --- | --- | --- | --- | --- | --- | --- | --- | --- | --- |
| ICA  D = 200  N=819 | Nothing  Amps & spatial  Connectivity only  Amplitudes only  Spatial maps only | -  -  **✓**  -  - | -  **✓**  -  **✓**  - | -  **✓**  -  -  **✓** | -0.0003  0.61  0.02*  0.08  0.53 | 0.02  0.58  0.06*  0.12  0.51 | 0.65  0.77  0.66*  0.68  0.76 | 0.18364  0.00001  0.0417*  0.00109  0.00001 | 0.15  0.78  0.14*  0.42  0.77 |
| ICA  D = 25  N=819 | Nothing  Amps & spatial  Connectivity only  Amplitudes only  Spatial maps only | -  -  **✓**  -  - | -  **✓**  -  **✓**  - | -  **✓**  -  -  **✓** | -0.001  0.96  0.56  0.15  0.73 | -0.02  0.50  0.38  0.11  0.65 | 0.64  0.71  0.69  0.67  0.70 | 0.48333  0.00001  0.00007  0.03009  0.00001 | 0.11  0.54  0.24  0.39  0.50 |
| Yeo parcellation  D = 109  N=819 | Nothing  Amps & spatial  Connectivity only  Amplitudes only  Spatial maps only | -  -  **✓**  -  - | -  **✓**  -  **✓**  - | -  **✓**  -  -  **✓** | 0.0007  0.91  0.09*  0.11  0.75 | 0.02  0.29  0.29*  0.001  0.72 | 0.64  0.73  0.67*  0.68  0.76 | 0.57151  0.00001  0.0192*  0.00312  0.00001 | 0.15  0.53  0.35*  0.39  0.63 |
| HCP_MMP1.0 subject parcellation  D = 360  N=441 | Nothing  Amps & spatial  Connectivity only  Amplitudes only  Spatial maps only | -  -  **✓**  -  - | -  **✓**  -  **✓**  - | -  **✓**  -  -  **✓** | 0.27  0.81  0.30*  0.25  0.73 | 0.40  0.36  0.44*  0.03  0.79 | 0.85  0.87  0.86*  0.85  0.88 | 0.04779  0.00038  0.0064*  0.06235  0.00001 | 0.36  0.58  0.37*  0.46  0.55 |
| HCP_MMP1.0 group parcellation  D = 360  N = 441 | Nothing  Amps & spatial  Connectivity only  Amplitudes only  Spatial maps only | -  -  **✓**  -  - | -  **✓**  -  **✓**  - | -  **✓**  -  -  **✓** | -0.0003  0.76  0.07*  0.05  0.68 | -0.05  0.33  0.36*  -0.02  0.74 | 0.84  0.87  0.85*  0.84  0.87 | 0.11041  0.00018  0.0491*  0.22268  0.00005 | 0.17  0.60  0.20*  0.26  0.59 |

**Supplementary file 1e**

|  | **Spatial maps** | **Z_network matrix_** | **R_correlation_** | **CCA r_U-V_** | **CCA P_U-V_** | **CCA r_U-Uica_** |
| --- | --- | --- | --- | --- | --- | --- |
| ICA 200 Full | Thresholded  Binarised  Binarised % | 0.43  0.45  0.44 | 0.32  0.43  0.44 | 0.74  0.74  0.70 | 0.00001 0.00001  0.00001 | 0.67  0.63  0.57 |
| ICA 200  Partial | Thresholded  Binarised  Binarised % | 0.29  0.28  0.41 | 0.42  0.54  0.55 | 0.74  0.76  0.77 | 0.00001 0.00001  0.00001 | 0.73  0.77  0.76 |
| Yeo 109  Full | Thresholded  Binarised  Binarised % | 0.57  0.56  0.44 | 0.37  0.37  0.37 | 0.71  0.71  0.72 | 0.00001 0.00001  0.00001 | 0.56  0.54  0.56 |
| Yeo 109  Partial | Thresholded  Binarised  Binarised % | 0.48  0.46  0.54 | 0.68  0.62  0.62 | 0.75  0.75  0.75 | 0.00001 0.00001  0.00001 | 0.63  0.67  0.52 |
