# Supplementary material for: The relationship between spatial configuration and functional connectivity of brain regions
Source: eLife. 2018 Feb 16;7:e32992. doi: 10.7554/eLife.32992 (PMC5860869; doi:10.7554/eLife.32992)
Supplement: Transparent reporting form [file elife-32992-transrepform.docx]

***eLife’s* transparent reporting form**

We encourage authors to provide detailed information *within their submission* to facilitate the interpretation and replication of experiments. Authors can upload supporting documentation to indicate the use of appropriate reporting guidelines for health-related research (see [EQUATOR Network](http://www.equator-network.org/%20)), life science research (see the [BioSharing Information Resource](https://biosharing.org/)), or the [ARRIVE guidelines](http://www.plosbiology.org/article/info:doi/10.1371/journal.pbio.1000412) for reporting work involving animal research. Where applicable, authors should refer to any relevant reporting standards documents in this form.

If you have any questions, please consult our Journal Policies and/or contact us: [editorial@elifesciences.org](mailto:editorial@elifesciences.org).

**Sample-size estimation**

- You should state whether an appropriate sample size was computed when the study was being designed
- You should state the statistical method of sample size computation and any required assumptions
- If no explicit power analysis was used, you should describe how you decided what sample (replicate) size (number) to use

Please outline where this information can be found within the submission (e.g., sections or figure legends), or explain why this information doesn’t apply to your submission:

This manuscript makes use of publicly available large-scale datasets (i.e. the Human Connectome project). For the majority of analysis all 820 subject available at the time of this work were used. Previous work has successfully adopted the canonical correlation methods used here in approximately half the subjects (Smith, S. M., Nichols, T. E., Vidaurre, D., Winkler, A. M., Behrens, T. E. J., Glasser, M. F., … Miller, K. L. (2015). A positive-negative mode of population covariation links brain connectivity, demographics and behavior. *Nature Neuroscience*. https://doi.org/10.1038/nn.4125)

**Replicates**

- You should report how often each experiment was performed
- You should include a definition of biological versus technical replication
- The data obtained should be provided and sufficient information should be provided to indicate the number of independent biological and/or technical replicates
- If you encountered any outliers, you should describe how these were handled
- Criteria for exclusion/inclusion of data should be clearly stated
- High-throughput sequence data should be uploaded before submission, with a private link for reviewers provided (these are available from both GEO and ArrayExpress)

Please outline where this information can be found within the submission (e.g., sections or figure legends), or explain why this information doesn’t apply to your submission:

The simulation approach was replicated using multiple parcellations (i.e. ICA at a dimensionality of 25 and 200, Yeo parcellation, and HCP_MMP 1.0 parcellation) and multiple different dimensionalities (ranging from 25 to 360). For the full set of results, please see Supplementary file 1.

Additionally, to ensure that our results were not driven by effects that are specific to the PROFUMO decomposition, we performed an entirely independent analysis based on task contrast maps. The results of this independent analysis are presented in Figure 5.

**Statistical reporting**

- Statistical analysis methods should be described and justified
- Raw data should be presented in figures whenever informative to do so (typically when N per group is less than 10)
- For each experiment, you should identify the statistical tests used, exact values of N, definitions of center, methods of multiple test correction, and dispersion and precision measures (e.g., mean, median, SD, SEM, confidence intervals; and, for the major substantive results, a measure of effect size (e.g., Pearson's r, Cohen's d)
- Report exact p-values wherever possible alongside the summary statistics and 95% confidence intervals. These should be reported for all key questions and not only when the p-value is less than 0.05.

Please outline where this information can be found within the submission (e.g., sections or figure legends), or explain why this information doesn’t apply to your submission:

In general, permutation testing (100,000 permutations) was used to assess the significance of canonical correlation results, as explained on page 33 on the main manuscript. Additionally, surrogate data were generated to obtain confidence intervals of some results, as explained on page 34 of the main manuscript.

Subject numbers (N) are explicitly defined in all tables and figures in the main manuscript and in the supplementary files.

(For large datasets, or papers with a very large number of statistical tests, you may upload a single table file with tests, Ns, etc., with reference to sections in the manuscript.)

**Group allocation**

- Indicate how samples were allocated into experimental groups (in the case of clinical studies, please specify allocation to treatment method); if randomization was used, please also state if restricted randomization was applied
- Indicate if masking was used during group allocation, data collection and/or data analysis

Please outline where this information can be found within the submission (e.g., sections or figure legends), or explain why this information doesn’t apply to your submission:

This work does not involve any experimental groups.

**Additional data files (“source data”)**

- We encourage you to upload relevant additional data files, such as numerical data that are represented as a graph in a figure, or as a summary table
- Where provided, these should be in the most useful format, and they can be uploaded as “Source data” files linked to a main figure or table
- Include model definition files including the full list of parameters used
- Include code used for data analysis (e.g., R, MatLab)
- Avoid stating that data files are “available upon request”

Please indicate the figures or tables for which source data files have been provided:

Data for many of the figures will be shared on Balsa upon publication: <https://balsa.wustl.edu/study/show/kKM0>. Matlab code used for data analysis can be found here: <https://github.com/JanineBijsterbosch/Spatial_netmat>.
